# Analysis of the miRNA Transcriptome in *Aconitum vilmorinianum* and Its Regulation of Diterpenoid Alkaloid Biosynthesis

**DOI:** 10.3390/ijms26010348

**Published:** 2025-01-03

**Authors:** Xing Zhao, Yiguo Li, Jun Shen, Caixia Guo, Jie Li, Mingzhu Chen, Huini Xu, Kunzhi Li

**Affiliations:** Faculty of Life Science and Technology, Kunming University of Science and Technology, Kunming 650500, China

**Keywords:** *Aconitum vilmorinianum*, miRNAs, diterpenoid alkaloids, target gene

## Abstract

*Aconitum vilmorinianum* (*A. vilmorinianum*) is an important medicinal plant in the Aconitum genus that is known for its diterpenoid alkaloids, which exhibit significant pharmacological activity and toxicity, thus making it valuable for both medicinal use and as a biopesticide. Although the biosynthesis of terpenoids is well characterized, the potential gene regulatory role of microRNAs (miRNAs) in terpenoid biosynthesis in *A. vilmorinianum* remains unclear, and further research is needed to explore this aspect in this species. In this study, miRNA sequencing was conducted to analyze the miRNA population and its targets in *A. vilmorinianum*. A total of 22,435 small RNAs were identified across the nine samples. Through miRNA target gene association analysis, 356 target genes from 54 known miRNAs and 977 target genes from 151 novel miRNAs were identified. Target identification revealed that miR6300 targets the hydroxymethylglutaryl-CoA reductase (*HMGR*) gene, which is involved in the formation of the terpenoid backbone and regulates the synthesis of diterpenoid alkaloids. Additionally, preliminary findings suggest that miR4995 and miR5021 may be involved in the regulation of terpenoid biosynthesis, although further biochemical analysis is needed to confirm these potential roles. This study provides a foundational understanding of the molecular mechanisms by which miRNAs regulate terpenoid biosynthesis in *A. vilmorinianum* and offers scientific evidence for further research on the biosynthesis of diterpenoid alkaloids in this medicinal plant.

## 1. Introduction

*Aconitum vilmorinianum* (*A. vilmorinianum*) is a species of the *Aconitum* genus in the Ranunculaceae family. Globally, there are 350 species in the *Aconitum* genus, with over 200 species found in China, including 66 species, 25 varieties, and four forms observed in Yunnan, which is the center of *Aconitum* distribution [1,2]. Aconitine, which is a diterpenoid alkaloid, is a type of terpenoid that plays a crucial role in plant growth, development, and the synthesis of secondary metabolites. Many terpenoid compounds have significant pharmacological activities and are widely used [1]. *A. vilmorinianum* is one of the most important medicinal plants in the *Aconitum* genus. Its primary active components include diterpenoid alkaloids, which are also responsible for its toxicity, thus making it valuable as both a medicinal herb and a biopesticide. Chemical studies on *A. vilmorinianum* have identified over 40 diterpenoid alkaloids, including vilmorrianine, aconitine A, and mesaconitine A, which have demonstrated efficacy in treating rheumatism, dispelling cold, promoting blood circulation, and exerting antitumor effects [3].

Research on terpenoid metabolic pathways in plants such as ginseng, carrot, osmanthus, and camphor [4,5,6,7] indicates that terpenoids are a diverse and functionally rich class of secondary metabolites. The biosynthetic pathways of diterpenoid alkaloids in *A. vilmorinianum* primarily include the mevalonate (MVA) pathway, the methylerythritol 4-phosphate (MEP) pathway, and the farnesyl diphosphate synthase (FDPS) pathway, which together form the diterpenoid backbone, thus eventually leading to the production of aconitine and its derivatives [8]. Although the metabolic pathways that are involved in the synthesis of diterpenoid alkaloids in *A. vilmorinianum* are known, the miRNAs that regulate alkaloid synthesis have not yet been identified.

MiRNAs have a wide range of functions in plants and participate in the regulation of various physiological and biochemical processes, including plant growth and development, morphogenesis, signal transduction, and hormone secretion [9]. In crops, fruits, flowers, and medicinal plants, modern bioinformatics, sequencing technologies, and gene cloning methods have been used to identify many miRNAs [10,11]. Some of these miRNAs are involved in regulating the synthesis of terpenoids. For example, the upstream biosynthesis pathways of ginsenosides, such as the MVA and MEP pathways, have been investigated through miRNAome sequencing, thus revealing 73 known miRNAs and 28 novel miRNAs and identifying a set of miRNAs associated with the accumulation of secondary metabolites [7]. In Taxus spp., paclitaxel (which is a diterpenoid alkaloid) has been studied, thus resulting in the identification of 871 miRNAs and 37 novel miRNAs, among which miR164 regulates the 13α-hydroxylase gene and miR171 regulates the taxadiene 2α-O-benzoyltransferase gene, both of which influence paclitaxel biosynthesis [12,13]. Additionally, miRNA156 is known to regulate anthocyanin and flavonoid biosynthesis [14], whereas miR156-SPL9 modulates sesquiterpene biosynthesis in *Pogostemon cablin* [15]. However, the mechanisms by which miRNAs regulate terpenoid biosynthesis in *A. vilmorinianum* remain unclear. Therefore, it is necessary to further investigate the role of miRNAs in regulating the biosynthesis and accumulation of terpenoids in *A. vilmorinianum*.

In this study, we performed miRNAome sequencing analysis on *A. vilmorinianum* using the BGISEQ-500 platform and conducted high-throughput sequencing of root tissues during the early, middle, and late stages of tuber formation. The aim of this study was to identify known and novel miRNAs. Based on miRNA target gene prediction, Eukaryotic Orthologous Group (KOG), Gene Ontology (GO), and Kyoto Encyclopedia of Genes and Genomes (KEGG) pathway analyses were performed to obtain functional annotations, thus identifying the miRNAs and their target genes that are involved in the biosynthesis of alkaloids in *A. vilmorinianum*. This study elucidates the molecular mechanisms by which miRNAs regulate alkaloid synthesis in *A. vilmorinianum*, which can provide a theoretical basis for the gene regulation of alkaloid synthesis in *A. vilmorinianum* and other *Aconitum* species.

## 2. Results

### 2.1. Overview of miRNA Sequencing

The types and quantities of small RNA fragments were statistically analyzed from the sequencing data of nine small RNA samples derived from the tuberous roots of *A. vilmorinianum* at the early, middle, and late stages of root formation (Supplementary Table S2). The results indicated that the number of small RNA fragments in these nine samples ranged from a minimum of 27,806,198 to a maximum of 30,188,472. After removing adapters, contaminants, and poly A sequences, the number of clean reads ranged from a minimum of 22,019,349 to a maximum of 28,421,521 (Supplementary Table S2). The sequencing results of the nine small RNA samples (Supplementary Table S3) revealed that the number of known miRNAs ranged from a minimum of 48 to a maximum of 54; moreover, the number of novel miRNAs ranged from 130 to 151.

**Figure 1:**
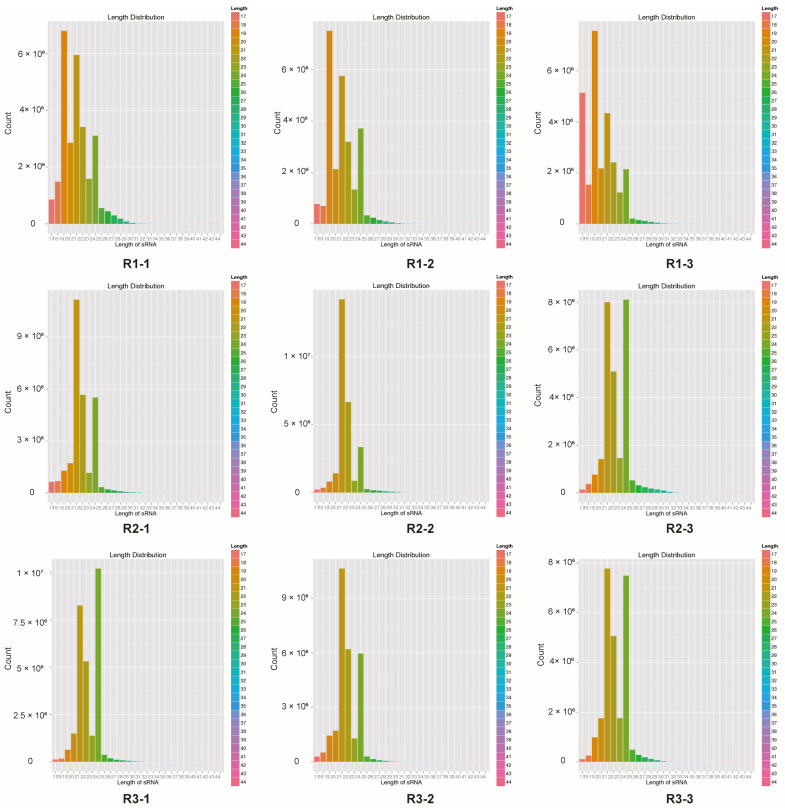
Length distribution of sRNA sequences in each sequencing sample. The samples were collected at three different stages: the initial stage of root formation in June (**R1**), the middle stage in August (**R2**), and the final stage in October (**R3**). Each stage had three biological replicates (labeled as (**R1-1**–**R3-3**)).

The proportion of low-quality bases (<20) in the nine miRNA samples was relatively low, thus indicating that the overall sequencing quality was high (Supplementary Figure S1). The overall fragment distribution of the nine miRNA samples (Figure 1) revealed that the sequenced fragment lengths were concentrated in the range of 18 nt to 30 nt, thus suggesting that the sample quality was good and suitable for subsequent miRNA analysis and research.

The sequencing results were subjected to BLAST analysis against small RNA databases, following the order of miRNA, piRNA, snoRNA, Rfam, and other sRNAs for annotation. According to the annotation results (Supplementary Table S4), across the nine samples, the number of sequences that could be aligned with the small RNA database ranged from a minimum of 8,949,882 to a maximum of 18,589,476. The proportion of sequences in each sample that could be aligned with the database was greater than 40.65%, with the highest percentage reaching 72.42%.

### 2.2. Identification of Known and Novel miRNAs

By aligning the sequences with the database, the results revealed a total of 54 known miRNAs across the nine samples (Supplementary Table S5). Among the three samples obtained from the early stage of root formation, 50 known miRNAs were identified. Among these genes, miR168a-5p, miR168a-3p, miR166a, miR166a-3p, and miR159b-3p were highly expressed, with miR168a-5p showing the highest expression level at 26,898 reads. In contrast, miR3630-3p-1, miR828a-1, miR390-1, miR5368, miR530-3, and miR156k exhibited low expression levels, with each of these genes exhibiting fewer than 10 reads.

During the middle stage, 54 known miRNAs were identified across the three samples. Among them, miR160b-1, miR159b-3p, miR171a-3p, miR168a-5p, miR168a-3p, and miR166h-3p were highly expressed, with miR159b-3p demonstrating the highest expression level at 193,097 reads.

In the late stage of root formation, 47 known miRNAs were identified across the three samples, with miR168a-3p, miR159b-3p, miR319a-1, and miR168a-5p being highly expressed. The highest expression level was observed for miR159b-3p at 367,940 reads.

**Figure 2:**
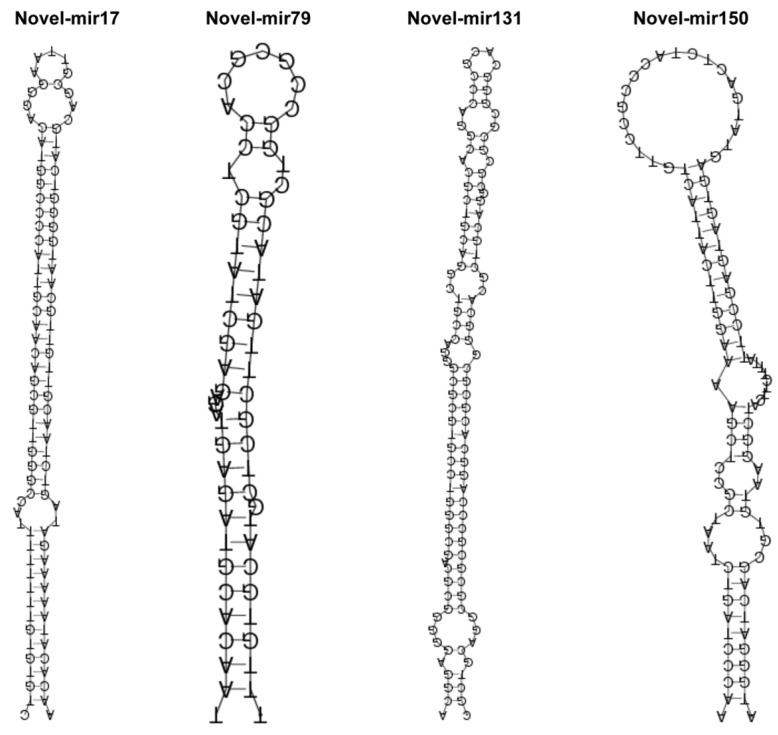
Stem-loop hairpin structures of Novel-mir17, Novel-mir79, Novel-mir131, and Novel-mir150.

After mature miRNAs were identified through alignment with the miRNA database, the remaining unmatched sequences were subjected to novel miRNA prediction. Based on the characteristics of known miRNAs, potential miRNAs were predicted, thus resulting in the identification of 45,133 small RNAs and 439,603 unknown small RNAs. For the 151 novel miRNAs with annotated target gene information (Supplementary Table S6), secondary structure prediction was performed using RIPmiR 12.0 (Figure 2) [16].

### 2.3. Differential Expression Analysis of miRNAs

**Figure 3:**
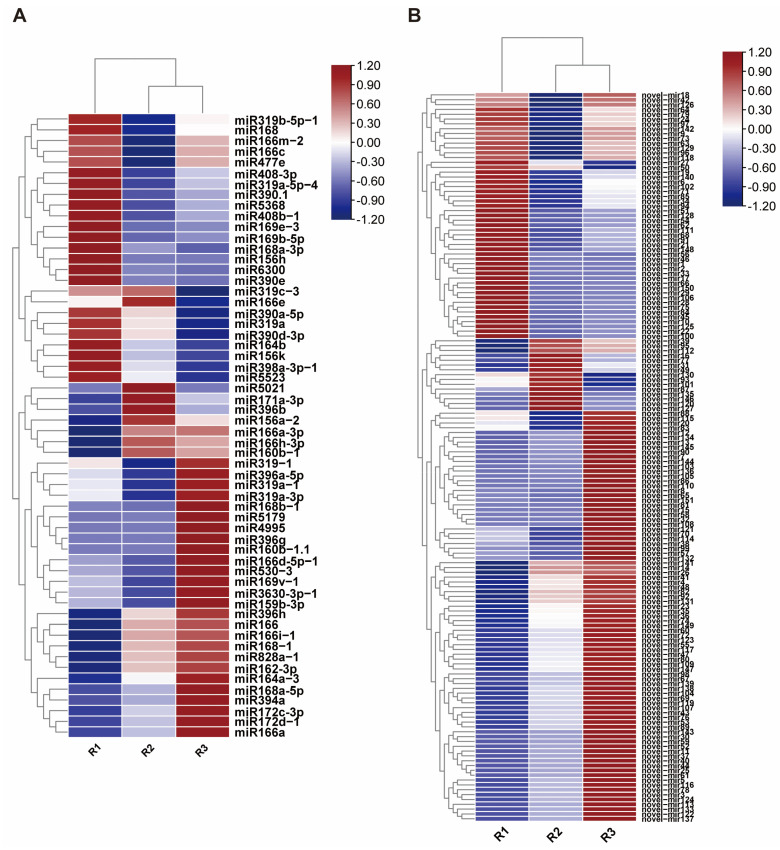
Expression patterns of mature miRNAs and novel miRNAs at the initial, middle, and final stages of root formation. (**A**) The expression levels of known miRNAs. (**B**) The expression levels of novel miRNAs.

The expression levels of known miRNAs and novel miRNAs were normalized using TPM. The results (Figure 3, Supplementary Tables S7 and S8) indicated that among the miRNAs regulating terpenoid synthesis, miR6300 had high expression levels during the early stage of tuber formation, with a decrease in expression being observed during the middle and late stages. The expression level of miR168a-5p increased as tuber formation progressed. miR159b-3p was highly expressed throughout the tuber formation process, with the highest expression occurring at the early stage, followed by a decrease in the middle stage. The expression of miR166a-3p increased as tuber formation progressed. The expression levels of miR828a_1, miR390_1, miR156h, miR5368, miR5523, miR530_3, and miR156k were very low, and miR396g and miR160b_1 were only expressed at trace levels during the late stage, whereas miR5021 was only expressed at trace levels during the middle stage. Among the novel miRNAs, although their target genes have annotation information, none were found to be involved in the regulation of diterpenoid alkaloids.

**Figure 4:**
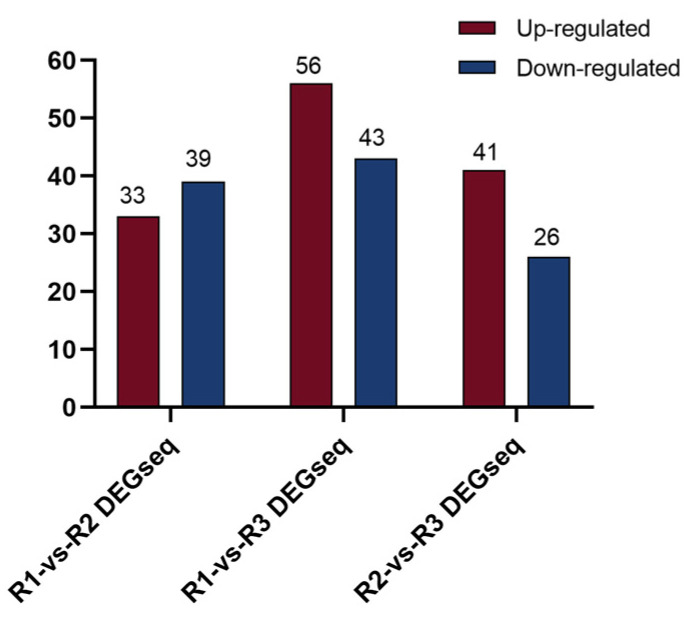
Differentially expressed miRNAs at three developmental stages. Blue and green indicate the numbers of downregulated and upregulated miRNAs, respectively.

Through comparative analysis of miRNA expression levels at different stages of tuber formation in *A. vilmorinianum*, significantly differentially expressed miRNAs were identified. The results (Figure 4) indicated that, compared with those in the early stage of tuber formation, 72 miRNAs with significantly different expression levels were expressed in the middle stage, with 33 miRNAs being upregulated and 39 miRNAs being downregulated. A comparison of the early and late stages revealed that 99 miRNAs were significantly differentially expressed, with 56 miRNAs being upregulated and 43 miRNAs being downregulated. Between the middle and late stages, 67 miRNAs were differentially expressed, with 41 upregulated miRNAs and 26 downregulated miRNAs being demonstrated.

### 2.4. miRNA Target Gene Prediction and Target Gene Annotation

To more accurately predict the potential target genes of miRNAs, we used psRobot v 1.2 and TargetFinder v 5.8 software for miRNA target prediction [17,18]. The statistical results of the number of target genes predicted by the different software programs indicated that TargetFinder software predicted 1601 target genes, whereas psRobot software predicted 1356 target genes, 557 of which were predicted by both software programs (Supplementary Figure S2).

Among the 54 known miRNAs, there were 356 corresponding target genes, whereas 151 novel miRNAs were found to regulate 977 target genes. Among the target genes of seven novel miRNAs, 48 genes had functional annotations (Supplementary Table S9). These seven novel miRNAs included Novel-mir121, Novel-mir102, Novel-mir101, Novel-mir23, Novel-mir16, Novel-mir2, and Novel-mir1 (Supplementary Table S9).

### 2.5. GO and KEGG Analysis

**Figure 5:**
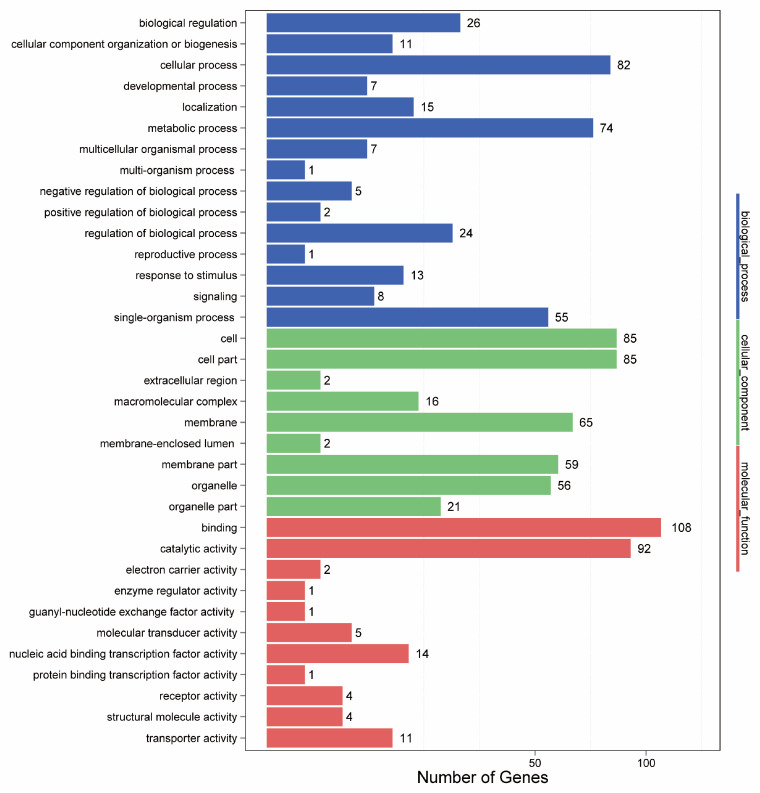
Gene ontology (GO) analysis of the target genes of the differentially expressed miRNAs.

Based on the predicted target genes of the differentially expressed miRNAs, GO functional classification was performed. The results (Supplementary Table S10, Figure 5) revealed that the annotation information of 965 target genes was categorized into three major categories and 36 subcategories. Among the three major categories, the biological process category included 331 target genes, the cellular component category included 391 target genes, and the molecular function category included 243 target genes. In the biological process category, the largest number of target genes were associated with cellular processes (82 genes), metabolic processes (74 genes), and single-organism processes (55 genes), whereas the smallest number of genes were associated with multi-organism processes (one gene) and reproductive processes (one gene). In the cellular component category, target genes were most abundant in cell components (85 genes), cell parts (85 genes), and cell membranes (65 genes), with the fewest genes observed in extracellular regions (two genes). In the molecular function category, target genes were most prevalent in binding activities (108 genes), catalytic activities (92 genes), nucleic acid binding transcription factor activities (14 genes), and transport activities (11 genes), whereas the least prevalent genes were found in enzyme regulator activities (one gene), guanine nucleotide exchange factor activities (one gene), and protein binding transcription factor activities (one gene). We focused on target genes that were related to diterpenoid alkaloid metabolism, which were distributed in the metabolic process category within the biological process category, with a total of 74 target genes being observed.

**Figure 6:**
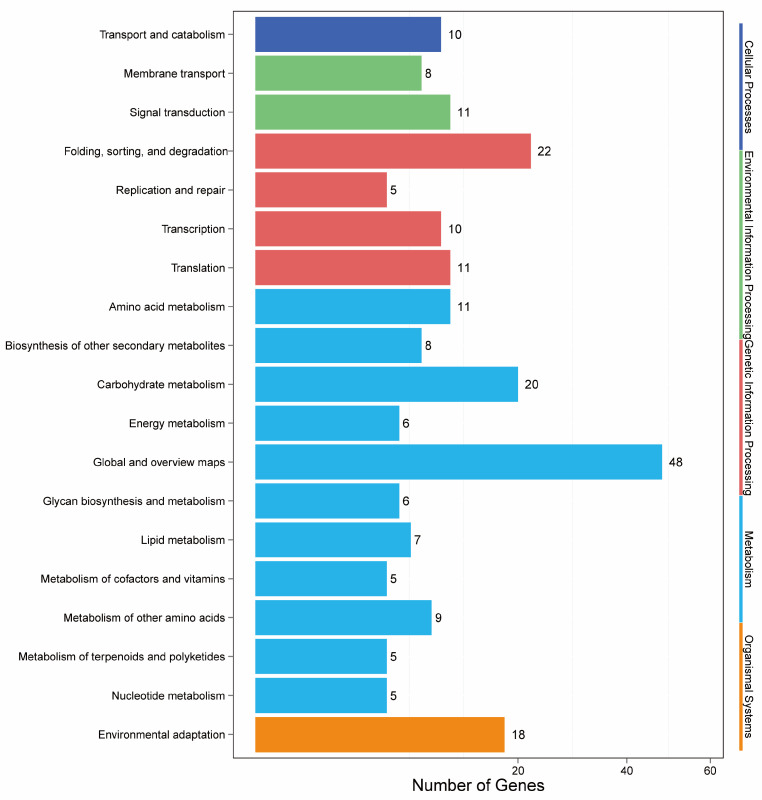
Kyoto Encyclopedia of Genes and Genomes (KEGG) analysis of target genes of differentially expressed miRNAs.

Based on the predicted target genes of the differentially expressed miRNAs, pathway enrichment analysis was performed by comparison with the KEGG database to identify the major metabolic pathways that were involved. The results revealed that 214 target genes were annotated by the KEGG database. After classification, these target genes were divided into five major categories and 19 subcategories. The five major categories included cell processes (5), environmental information processing (24), genetic information processing (49), metabolism (126), and organic systems (10) (Supplementary Table S11, Figure 6). Among the 19 subcategories, target genes were most prevalent in global and overview maps (48 genes), signal transduction (18 genes), and degradation, classification, and folding (18 genes), whereas the fewest target genes were associated with polysaccharide biosynthesis and metabolism (two genes). We focused on target genes that were predicted to be related to diterpenoid alkaloids, which were demonstrated to be involved in terpenoid and polyketide metabolism, with a total of four target genes being observed. This annotation helps to enhance research into the diterpenoid alkaloid metabolism process and can assist in identifying target genes that are involved in this type of metabolism. Information on these miRNAs is also an important resource for promoting diterpenoid alkaloid accumulation in future molecular breeding efforts.

### 2.6. Identification of miRNAs That Regulate Diterpenoid Alkaloid Biosynthesis

Based on the KEGG functional analysis of the target genes, we identified miRNAs and their target genes that were involved in the regulation of terpenoid synthesis in *A. vilmorinianum* (Table 1). The miRNAs that were implicated in the regulation of terpenoid synthesis include miR6300, miR396a-5p, and miR166d-5p-1. The target genes regulated by miR6300 included Unigene66964-All and Unigene17174-All; those genes regulated by miR396a-5p included CL11821.Contig1-All and Unigene40355-All; and the target gene regulated by miR166d-5p-1 included Unigene6401-All.

### 2.7. Validation of miRNA Expression by qRT-PCR

**Figure 7:**
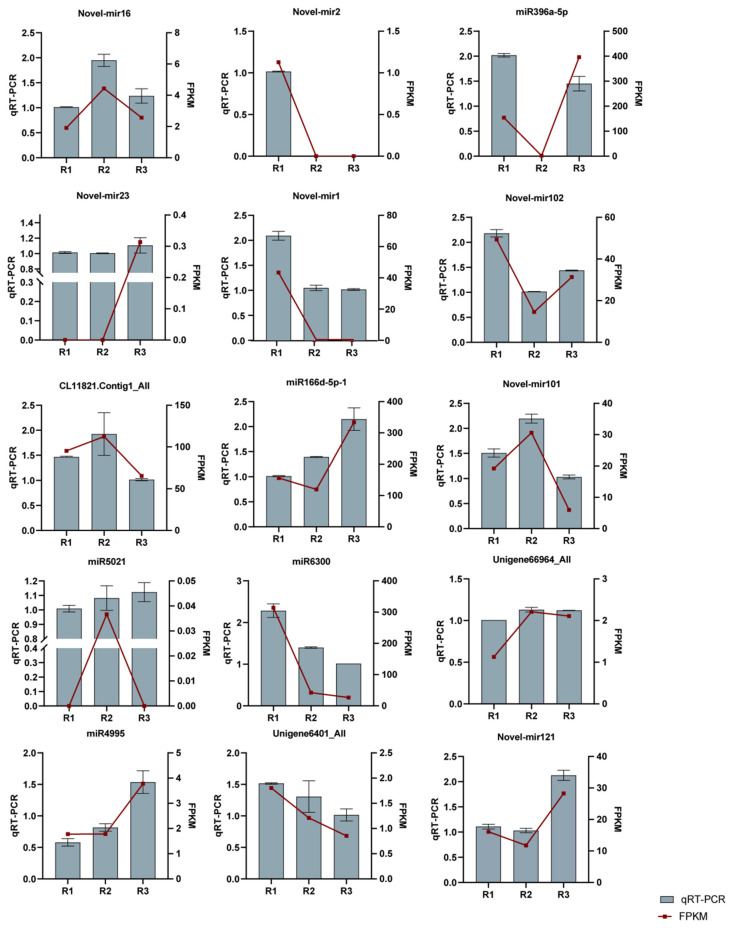
Validation of the expression of 15 differentially expressed miRNAs at three root developmental stages by qRT-PCR. 18S rRNA was used as an internal reference for normalization by qRT-PCR. All of the qRT-PCR reactions were performed with three biological and three technical replications.

To validate the sRNA sequencing results, five mature miRNAs, seven novel miRNAs, and three target genes were selected for qRT-PCR analysis (Figure 7). The results revealed that the expression trends of the 12 selected miRNAs and three target genes obtained by qRT-PCR were similar to those of the sRNA sequencing data, thus confirming the reliability of the sRNA sequencing data.

## 3. Discussion

Recent studies have increasingly highlighted the important role of miRNAs in regulating biosynthesis, particularly in plant secondary metabolism [19,20,21]. However, the potential gene regulatory roles of miRNAs in *A. vilmorinianum* remain unclear. Therefore, this study used miRNA sequencing to explore the potential regulatory network of miRNAs and the mechanisms controlling terpenoid synthesis in *A. vilmorinianum* roots.

Analysis of the length distribution of the sRNA sequences revealed that 19–24 nt was the most common sRNA length (Figure 1), which is consistent with previous reports on other medicinal plants [22,23,24]. We identified 54 known miRNAs, 151 novel miRNAs, and 22,435 novel small RNAs, with no piRNAs being detected (Supplementary Tables S5 and S6). The stem-loop hairpin structures of four randomly selected *A. vilmorinianum* miRNAs are shown in Figure 2. The novel miRNAs ranged in length from 19 to 30 nt. It has been shown that miRNAs regulate plant growth and development processes by regulating the expression of target genes. The identification of miRNA target genes is crucial for revealing the regulatory role of miRNAs [9]. Among the 54 known miRNAs, 214 target genes were found to be regulated and involved in processes such as cell growth and development, transcriptional regulation, signal transduction, and secondary metabolism (Supplementary Table S11). Due to their extensive biological activities, secondary metabolites that are produced by plants not only play crucial roles in plant growth, development, and environmental adaptation but also serve as important raw materials in the pharmaceutical industry [25]. GO analysis revealed that 74 target genes were distributed within the metabolic process category of biological processes (Figure 5). The KEGG database annotated 126 target genes related to metabolism (Figure 5). These findings suggest that the growth and development of *A. vilmorinianum* roots are regulated by a variety of miRNAs, with key roles played by genes primarily involved in metabolic processes. Compared with plants such as *Panax ginseng* and *Taxus*, *A. vilmorinianum* has fewer known miRNAs. For example, *Panax ginseng* research has identified 73 known miRNAs and 28 novel miRNAs, which are related to the accumulation of secondary metabolites [7]. In *Taxus*, 37 novel miRNAs and 871 known miRNAs were found, with miR164 regulating taxane 13α-hydroxylase and miR171 regulating taxadiene 2α-O-benzoyltransferase, thus influencing Taxol biosynthesis [13]. In *Taxus wallichiana*, miR172a, miR156a, miR396b, miR168a, miR169b, miR480, and miR1310 were identified as being involved in terpenoid biosynthesis [12]. Additionally, miRNA156 regulates anthocyanin and flavonoid biosynthesis [14], whereas miR156-SPL9 regulates sesquiterpene biosynthesis in *patchouli* [15]. Furthermore, the majority of the miRNAs that were identified in this study were not annotated, which is likely due to the lack of genomic information for *A. vilmorinianum*. Consequently, many novel and functionally specific miRNAs may still be present, thus suggesting that much information regarding this scenario remains to be explored.

The diterpenoid synthesis pathway in *A. vilmorinianum* includes the MVA pathway, the MEP pathway, the FDPS regulatory pathway, and the diterpenoid skeleton synthesis pathway [8]. The MVA and MEP pathways produce terpenoid skeletons and generate precursors for diterpenoid biosynthesis. These precursors subsequently undergo modifications such as hydroxylation, glycosylation, methylation, and epoxidation, as well as processes involving cytochrome P450, dehydrogenation, reduction, glycosyl transfer, and methyl transfer to form diterpenoid alkaloids [26]. The biosynthetic pathway of the diterpenoid alkaloids in *A. vilmorinianum* is essentially understood; however, the regulation of this pathway remains unclear. This study conducted miRNA sequencing analysis during the root formation of *A. vilmorinianum* and preliminarily demonstrated the molecular regulatory mechanism of miRNAs in modulating the biosynthetic pathway of diterpenoid alkaloids in *A. vilmorinianum*. Studies have shown that miRNAs such as miR6300, miR5021, and miR4995 are involved in the regulation of terpenoid formation, as well as the production of other plant secondary metabolites [19]. The target genes of miR6300 are Unigene17174_All and Unigene66964_All. Unigene17174_All is annotated as cytochrome P450, which is involved in brassinosteroid biosynthesis. Unigene66964_All is annotated as the *HMGR* gene, which is involved in terpenoid backbone biosynthesis (Table 1). Previous transcriptome sequencing research in *A. vilmorinianum* roots identified the *HMGR* gene in the MVA pathway, which is a likely target of the miRNA–*HMGR*–miR6300 regulatory network [8]. In *Persicaria minor* and *Podophyllum hexandrum*, miR6300 was found to regulate the *HMGR* gene, which is involved in terpenoid synthesis [21,27]. Thus, the miRNA–*HMGR*–miR6300 network plays a crucial role in diterpenoid biosynthesis in *A. vilmorinianum*. In *Catharanthus roseus*, miR5021 regulates the main chain biosynthesis of terpenoids and affects the expression of geranylgeranyl diphosphate (*GGPP*) [28]. In *Xanthium strumarium*, miR5021 targets isopentenyl diphosphate isomerase (*IDI*), *HMGR*, and diphosphate synthase [29]. In *Cinnamomum camphora*, miR5021 targets geranylgeranyl diphosphate reductase (GGDR), which is involved in the downstream branch of the isoprenoid pathway and catalyzes the conversion of *GGPP* to pyrophosphate [6]. Chalcone synthase, which is another target gene of miR5021, regulates flavonoid synthesis in *Cinnamomum camphora* [6]. In *Sophora flavescens*, miR4995 targets 3-deoxy-D-arabino-heptulosonate 7-phosphate synthase, thus affecting the biosynthesis of sophoridine I through the regulation of terpenoid compounds [30]. In *A. vilmorinianum*, both miR5021 and miR4995 have been identified, with low expression of miR5021 and moderate expression of miR4995 being demonstrated. Further validation is needed to confirm their roles in regulating diterpenoid alkaloid synthesis.

From the miRNA sequencing data of *A. vilmorinianum*, we identified 151 novel miRNAs that regulate 977 target genes. Among the seven novel miRNAs, 48 target genes had functional annotations (Supplementary Table S9). Novel-mir2 regulates five target genes, three of which interact with plant pathogens, with another target gene being the MYC2 transcription factor, which is potentially involved in defense regulation. Novel-mir16 regulates 27 target genes, including 13 genes involved in protein kinases. Additionally, it regulates genes such as RNA polymerase, the disease resistance protein RPS2, histone demethylase, aldehyde reductase, and acetyl-CoA carboxylase. Novel-mir101 regulates three target genes, one of which is the cytochrome P450 reductase gene. Cytochrome P450 enzymes are key components in the biosynthesis and evolution of specialized metabolites, which exhibit various biological activities in the plant kingdom [31]. The biosynthesis of benzylisoquinoline alkaloids (BIAs) has numerous stages, thus necessitating the involvement of a suite of specific genes and enzymatic factors [31].

Key enzymes, such as 6-O-methyltransferase and P450 monooxygenases, are instrumental in the transformation of (S)-norcoclaurine into (S)-reticuline, which is a pivotal intermediate in the biosynthetic pathway of isoquinoline alkaloids. Subsequently, (S)-reticuline undergoes various modifications, thus leading to the generation of a variety of BIAs [20]. A study conducted by Xu et al. demonstrated that CPYP450 and tyrosine decarboxylase serve as targets of miR396, thus playing crucial roles in the biosynthetic pathway of alkaloids in *Lycoris aurea* [32]. These results indicate that Novel-mir101 may also be involved in regulating the biosynthesis of terpenoids in *A. vilmorinianum*. However, many novel miRNA target gene functions remain unclear, with these genes lacking homologs in other species. These genes may be found in *A. vilmorinianum* and perform a specific function; however, further analysis and research are needed to demonstrate these possible activities.

## 4. Materials and Methods

### 4.1. Plant Materials

Cultivated *A. vilmorinianum* was used as the experimental material. The plants were collected and cultivated at the experimental base of the School of Life Sciences and Technology, Kunming University of Science and Technology. The *A. vilmorinianum* plants used in this study were grown in soil. The tuberous roots of *A. vilmorinianum* were used as sample materials in this study. The samples were collected at three different stages: the initial stage of root formation in June (R1), the middle stage in August (R2), and the final stage in October (R3). Each stage had three biological replicates. After collection, the samples were stored at −80 °C for subsequent RNA extraction.

### 4.2. Small RNA Sample Preparation and Sequencing

Total RNA was extracted from the nine samples using a TRIzol reagent kit (Vazyme, Nanjing, China), after which any DNA contamination was removed. The purity and quality of the RNA were assessed, and samples meeting the quality standards were used to construct small RNA cDNA libraries for the nine samples. Small RNA molecules were separated using 1.5% denaturing polyacrylamide gel electrophoresis, and RNA fragments of 18–30 nt were collected from the samples. The constructed libraries were then sequenced using the BGISEQ-500 platform.

### 4.3. Bioinformatics Analysis of Sequencing Data 

After sequencing, adapters, contaminants, and low-quality sequences were removed, and the target sequences of 18–30 nt were screened and compared with the Rfam (http://www.sanger.ac.uk/Software/Rfam, accessed on 13 November 2022) and RepeatMasker (https://www.repeatmasker.org, accessed on 13 November 2022) databases to eliminate redundant sequences, ribosomal RNA, small nucleolar RNA, small nuclear RNA, transfer RNA, and protein-coding genes. The remaining clean sRNA sequences were aligned against the mature plant miRNAs in miRBase v22.0 [33] to identify known miRNAs (conserved miRNAs). Novel miRNAs in *A. vilmorinianum* roots were predicted using RIPmiR 12.0 software.

### 4.4. Identification of Differentially Expressed miRNAs

To determine the significance of differences in miRNA expression levels, the expression levels of miRNAs in the R1-1, R1-2, and R1-3; R2-1, R2-2, and R2-3; and R3-1, R3-2, and R3-3 samples were normalized by using transcripts per million (TPM) values. The formula for TPM calculation was applied to identify differentially expressed miRNAs between the initial, middle, and final stages of root formation in *A. vilmorinianum*. Furthermore, miRNAs with |log2 (fold change)| values greater than 1 and Q values less than 0.001 were considered to be significantly differentially expressed [34].

### 4.5. Target Gene Prediction and Functional Analysis

The target genes of the miRNAs were predicted using psRobot and TargetFinder software [17,18]. The intersection of the target genes that were predicted by both software programs was considered to be the final prediction result. These target genes were then compared with the unigenes obtained from transcriptome sequencing to identify the miRNA target genes. The target genes were annotated by comparison with the GO (http://www.Geneontology.org/, accessed on 13 November 2022) database and classified accordingly. Functional enrichment analysis was performed to identify the biological functions of the miRNAs corresponding to the target genes. The target genes were also compared with the KEGG (http://www.kegg.jp/kegg/, accessed on 13 November 2022) database for pathway enrichment analysis, thus identifying the metabolic pathways and functions that are regulated by the differentially expressed target genes.

### 4.6. qRT-PCR Validation

Five mature miRNAs (miR6300, miR166d-5p-1, miR396a-5p, miR5021, and miR4995) and seven novel miRNAs (Novel-mir121, Novel-mir102, Novel-mir101, Novel-mir23, Novel-mir16, Novel-mir2, and Novel-mir1) were selected, along with the following three target genes: Unigene66964_All (regulated by miR6300), Unigene6401_All (regulated by miR166d-5p-1), and CL11821.Contig1_All (regulated by miR396a-5p). The utilized qRT-PCR primers were designed based on the miRNA stem-loop sequences and gene sequences (Supplementary Table S1) [35], and qRT-PCR was subsequently performed. Total RNA was extracted, cDNA was synthesized according to the reverse transcription kit protocol, and qRT-PCR reactions were conducted using the PrimeScript RT Kit (Vazyme, Nanjing, China). 18S RNA was used as an internal control gene, and qRT-PCR was performed using the CFX96TM Real-Time System. The relative quantification method (2^−ΔΔCt^) was used to calculate the expression levels of the samples, and statistical analysis was conducted. Each sample was tested in triplicate [36].

## 5. Conclusions

Various miRNAs exist in medicinal plants, and these miRNAs play crucial regulatory roles in the biosynthesis of secondary metabolites in these plants. In this study, we identified and characterized 356 target genes for 54 known miRNA families and 977 target genes for 151 novel miRNAs in *A. vilmorinianum* roots. Target identification analysis revealed that miR6300 is involved in terpenoid skeleton formation and regulates diterpenoid alkaloid synthesis. Additionally, miR4995 and miR5021 may be related to the regulation of terpenoid compound biosynthesis. These findings enhance our understanding of the regulatory mechanisms underlying terpenoid biosynthesis in *A. vilmorinianum*.

## Figures and Tables

**Table 1:** miRNAs involved in diterpene synthesis and their target genes.

MiRNA	Differentially Expressed Genes	Category 1	Category 2	Metabolic Pathway
miR6300	Unigene17174_All	Metabolism	Metabolism of terpenoids and polyketides	Brassinosteroid biosynthesis
miR6300	Unigene66964_All			Terpenoid backbone biosynthesis
miR166d-5p-1	Unigene6401_All,			Carotenoid biosynthesis
miR396a-5p	Unigene40355_All			Carotenoid biosynthesis
miR396a-5p	CL11821.Contig1_All			Limonene and pinene degradation

## Data Availability

The miRNA transcriptome data for this study are available from the NCBI Sequence Read Archive (SRA) database with the following accession number: PRJNA1185462.

## Supplementary Materials

The supporting information can be downloaded at: https://www.mdpi.com/article/10.3390/ijms26010348/s1.
