# Supplementary material for: Analysis of the miRNA Transcriptome in Aconitum vilmorinianum and Its Regulation of Diterpenoid Alkaloid Biosynthesis
Source: Int J Mol Sci. 2025 Jan 3;26(1):348. doi: 10.3390/ijms26010348 (PMC11720529; doi:10.3390/ijms26010348)
Supplement: Supplementary file 1 [file ijms-26-00348-s001.zip › ijms-3396529-supplementary.pdf]

Figure S1 Base quality of each sequencing sample.

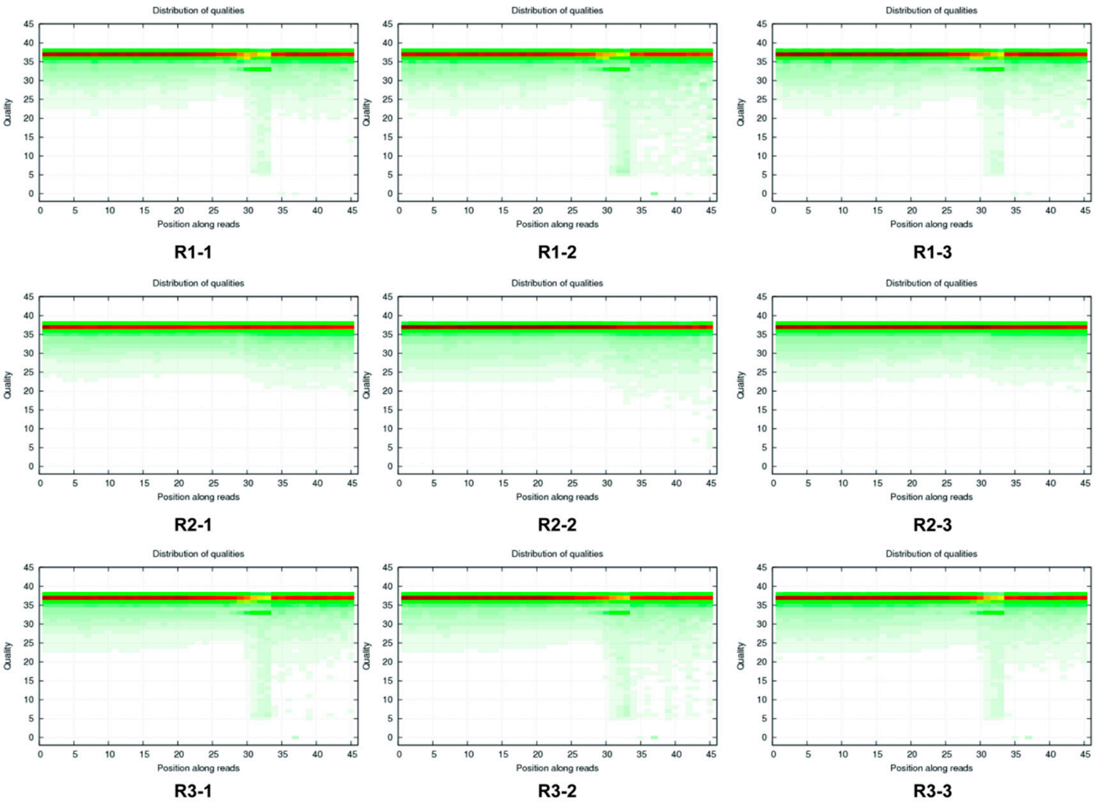

Figure S2 Wenn statistics of target gene prediction results.

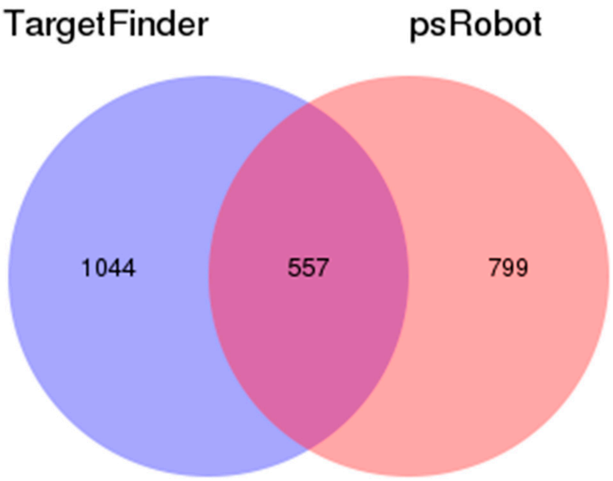

Supplementary Table S1 Primers sequences for 15 selected microRNAs (miRNAs) and reference gene (18S rRNA).

miRNA	Primer sequence	Annealing temperature	Product length
miR6300	F- CTGTCACTGCTGGGTCTG<br>R- GAGGTTGAGCCTTGGAAA	51.1<br>52.8	85
miR166d-5p-1	F- GACTTCGGACCAGGCTTCA<br>R-AACCCTTCATCATTACACCAATCT'	58.0<br>58.9	151
miR396a-5p	F- TTTTGCCCTATAAATACCC<br>R- TTGAAGCCCTCCTCGT	50.2<br>50.4	265
miR5021	F- TTGTTTGACCCACTTAT<br>R- TCAGACTGCTGCTTTT	51.8<br>52.1	163
miR4995	F- TGGTTAAGGGAACCCACC<br>R- TGAAGACTCGCTTTCGCTAC	55.0<br>55.8	44
Novel-mir1	F-TCGTGTTGAATGTCTGCACCTTCCT<br>R- CAACTCATCATCGCATCA	49.2<br>49.8	77
Novel-mir2	F- AGAGGCTTAGATGTGATTG<br>R- AGGGAGCCTGAGACAAA	49.6<br>49.8	54
Novel-mir16	F- AGGTGCTTCTTGATCTGTT<br>R- CCTTGTCGGTCTCCTAACT	51.3<br>50.2	107
Novel-mir23	F- CTTCTTCCATTATATCAAAATTTCA<br>R- TGAAATTTTGATATAATGGAAGAAG	49.3<br>50.5	168
Novel-mir101	F- AAGGGTGGTAATAATGAGGA<br>R- TGAGGAGCAGTGAAGGTC	51.4<br>49.9	251
Novel-mir102	F- TCCAGAGCGTTGGGTAT<br>CACTTGAGTGCCGTTAGA	52.3<br>51.9	127
Novel-mir121	F-GGGTGGGGTGGGTTTC<br>R-CGGTTTTGGGGCAGTT	55.1<br>54.1	113
Unigene66964_All	F-CTGGTAGCAGCAAGGATGT<br>R-GGAGGTATTATGAACGAGACCGCGT	53.1<br>53.1	148
Unigene6401_All	F- TCTCCCAAGCCGAACT<br>R- CACGGTAGACATACTCCTTT	50.4<br>49.2	240
CL11821.Contig1_All	F- GCGGATGGACCGTATC<br>R- CAGCCTCATGGAGCAG	50.9<br>49.8	199
18S rRNA	F- AGTTGGCTTCTTCGTTGT<br>R- CATCTTGGTAAACCTTGGTATAG	54.2<br>50.3	186

Supplementary Table S2 Summary of sequencing data for each sample.

Sample	Sequencing Type	Original Tag Count	Cleaned Tag Count	Proportion (%)
R1-1	SE50	29778314	26809821	90.03
R1-2	SE50	27818656	25211918	90.63
R1-3	SE50	29064753	22019349	75.76
R2-1	SE50	29978161	28022139	93.48
R2-2	SE50	30188472	28421521	94.15
R2-3	SE50	28152124	26811915	95.24
R3-1	SE50	29820026	28286670	94.86
R3-2	SE50	30151748	28309420	93.89
R3-3	SE50	27806198	26239192	94.36

Supplementary Table S3 Summary of detected smallRNA for each sample.

Sample	Known miRNA Count	Novel miRNA Count	Novel Small RNA Count
R1-3	51	130	9658
R2-3	52	140	12904
R3-3	54	138	8933
R1-1	51	134	12336
R2-2	53	131	16750
R3-2	48	142	13577
R2-1	53	143	22434
R3-1	50	140	10057
R1-2	52	138	9950

Supplementary Table S4 Alignment statistics of mapped tags.

Sample	Total Small RNA Count	Annotated Small RNA Count	Percentage (%)
R1-1	26809821	15365829	57.31
R1-2	25211918	11481725	45.54
R1-3	22019349	8949882	40.65
R2-1	28022139	18589476	66.34
R2-2	28421521	20581627	72.42
R2-3	26811915	15401776	57.44
R3-1	28286670	14592075	51.59
R3-2	28309420	17654229	62.36
R3-3	26239192	14186666	54.07

Supplementary Table S5 miRNAs and mature sequence.

miRNA Name	Mature Sequence	miRNA Name	Mature Sequence
miR3630-3p_1	TTTGGGAATCTCTCTGATGCAC	miR390a-5p	AAGCTCAGGAGGGATAGCGCC
miR398a-3p_1	TGTGTTCTCAGGTCACCCCTT	miR394a	TTGGCATTCTGTCCACCTCC
miR164b	TGGAGAAGCAGGGCACGTGC	miR166	TCGGACCAGGCTTCATTCCCCC
miR828a_1	TCTTGCTCAAATGAGTATTCCA	miR319c_3	CTTGGACTGAAGGGAGCTCCC
miR156a_2	TGACAGAAGAGAGAGAGCAC	miR160b_1	TGCCTGGCTCCCTGTATGCC
miR172d_1	AGAATCCTGATGATGCTGCAT	miR168a-3p	CCCGCCTTGCATCAACTGAAT
miR169e_3	AGCCAAGGATGACTTGCCGG	miR5523	TGAGGAGGAACATATTTACTAG
miR319_1	TTGGACTGAAGGGAGCTCC	miR166m_2	CGGACCAGGCTTCATTCCCC
miR6300	GTCGTTGTAGTATAGTGG	miR172c-3p	AGAATCTTGATGATGCTGC
miR168a-5p	TCGCTTGGTGCAGGTCGGGAA	miR396h	TCCACAGCTTTCTTGAACTG
miR390e	AGCTCAGGAGGGATAGCGCC	miR168	TCGCTTGGTGCAGGTCGGGAA
miR319a	CTTGGACTGAAGGGAGCTCC	miR390d-3p	CGCTATCCATCCTGAGTTTTA
miR166d-5p_1	GGAATGTTGTCTGGCTCGAGG	miR5179	TTTTGCTCAAGACCGCGCAAC

miR166h-3p	TCGGACCAGGCTTCATTCCC	miR408b_1	TGCACTGCCTCTTCCCTGGCT
miR319b-5p_1	AGAGCTCTCTTCAGTCCACTC	miR166a-3p	TCGGACCAGGCTTCATTCCCC
miR396a-5p	TTCCACAGCTTTCTTGAACTG	miR166a	TCGGACCAGGCTTCATTCC
miR390_1	TAAAGCTCAGGAGGGATAGCG	miR166c	CCGGACCAGGCTTCATCCCAG
miR5368	GGACAGTCTCAGGTAGACA	miR319a-3p	TTGGACTGAAGGGAGCTCCC
miR159b-3p	TTTGGATTGAAGGGAGCTCTT	miR396b	TTCCACAGCTTTCTTGAACT
miR408-3p	TGCACTGCCTCTTCCCTGCC	miR477e	CTCTCCCTCAAGGGCTTCTA
miR319a_1	TTGGACTGAAGGGAGCTCCCT	miR162-3p	TCGATAAACCTCTGCATCCA
miR171a-3p	TGATTGAGCCGCGCCAATATC	miR169v_1	CAGCCAAGGATGACTTGCC
miR168_1	ATTCAGTTGATGCAAGGCGGGATC	miR156k	TGACAGAAGAGAGAGAGCACA
miR4995	AGGCAGTGGCTTGGTTAAGGG	miR166i_1	TTGGACCAGGCTTCATTCCCC
miR319a-5p_4	GAGCTCTCTTCAGTCCACTC	miR166e	GGACCAGGCTTCATTCCCC
miR169b-5p	CAGCCAAGGATGACTTGCCGG	miR168b_1	TCGCTTGGTGCAGGTCGGG
miR164a_3	TGGAGAAGCAGGGCACGTGCA		

Supplementary Table S6 Novel miRNAs and mature sequence.

Novel miRNA	Mature Sequence	Novel miRNA	Mature Sequence
novel_mir1	CTAATATTTTTATGTCTGGACT	novel_mir77	GTTAAGTCAATCACAGGCACA
novel_mir2	CTCAGGCTCCCTTGTTTTT	novel_mir78	TCGTAATTTACAAGTTCGGGCCAA
novel_mir3	GACGGCGGCATCAATGTAGCGAGTAT	novel_mir79	CTCGCTTGATACGCTGGCCGCGCAC
novel_mir4	TAAGGGAGAGATGGTGAGCCTTGTGTTAGT	novel_mir80	GAGCCCGACCGTCGGATTACTTTATA
novel_mir5	ATGAGACAATTTGAAAGCCTGTAGCTCATT	novel_mir81	CTTCACCACTTGATGCTGCGGCA
novel_mir6	AACTATCGCACAAGGCCCAAG	novel_mir82	CGAATTCCAACGTTTAGGCCTC
novel_mir7	ACTATTTTACCCCGTGTATAATTGGTCAAA	novel_mir83	GTGTTTCGTGAAGGTTCAAACATAAAGACA
novel_mir8	CCTTCTGCCAAAATGTGGTCCTT	novel_mir84	AGTGGTTGTCAAGCACTTCCTG
novel_mir9	GGCTGATCGAGACCGGACGTAGAGCCACTA	novel_mir85	CAAGTTGTCCTTGGCTACATTGGGT
novel_mir10	CCTCAGACCGATGGCCAGACGGAGCGT	novel_mir86	TTGGCTCAATCATCCTCTACATTCGACAT
novel_mir11	CTGTATCTGCACTGTATCTGTGAC	novel_mir87	TAACTCGAAAGTGGTTCATGGAG
novel_mir12	GCAGAGGACTGTATCTGGGCCGCAGAGGA	novel_mir88	CCCGATGAACAAGTAAGAATTGCCTTAA

novel_mir13	TCAATGTGCTTACTCCTCTCGTGGT	novel_mir89	CATCTCATTGTGTCTCGTTCGGTTCAATCC
novel_mir14	GCCAAATCCGAATTGTCTAGACCCTTCTGT	novel_mir90	TGGATTGAACCGAACGAGACACAATGAGAT
novel_mir15	CAAGGGTGAGTGTTTGTAGGTAGATTAGT	novel_mir91	GTCTTGATCCGACTTGTCCTTTTTGGTA
novel_mir16	ACGGTTTCAAGGTGCTTCT	novel_mir92	GATTGAAGGGAGCTCTTCC
novel_mir17	GTTAAGGGACATGGCCCCATTGCAACAGCG	novel_mir93	AATTCCTAACGATAGTTGGAGACAT
novel_mir18	AGGAATAGTCATCTGATTAAGA	novel_mir94	TATTCGGAGTGATCATCGACA
novel_mir19	ATCCCAGTAGCCTCGTGCTGTGAAAACT	novel_mir95	GTGTGGCTGATCATCCTCT
novel_mir20	GTGGATGCAATGGAAAGTAT	novel_mir96	CCACACGAGCCCGGCTAGATTT
novel_mir21	GGTCCCTATCTGCCGTGGA	novel_mir97	AGTTGGGTTTGAGGGCATTTGCAATTTT
novel_mir22	GCATTGCTAGATACAACAGGTAT	novel_mir98	TGGGTGTTAAATGTTTTTTTGGA
novel_mir23	AGATACAACAGGTATCGTTGT	novel_mir99	GATTTTGGATTTGGCTCATTTTTGGTAGG
novel_mir24	CGTCGTCGCGCCTGCGGCGCT	novel_mir100	ATTAAGTATCTGAGGCTATCTTTTC
novel_mir25	CAGATTTTGGATTGGCGAAACTTATGAATA	novel_mir101	CGATTTGTGAAGCTCCTTCGAC
novel_mir26	ACTTGGAACGGGCAGATCAGAAATAAGCA	novel_mir102	TGGAGGGGAACGTATCCGGGTCCA
novel_mir27	GTTGAATAGTGTGGTTTGGGTGA	novel_mir103	TTTAGCAGTGAGTTCCTGACTC
novel_mir28	CAGGGCCTAAGCCACCCTGGACTAAGACTA	novel_mir104	TAGAGGAAGGATCGGATGACAAAG
novel_mir29	GCAAATGATGATGTTGTCCA	novel_mir105	CCCTCTGTTGTGGATACTAGGTTCACGG
novel_mir30	ACCGCTATGGCTTGCAAAATGTCCA	novel_mir106	TCAACAGCTTATAAGATGTTGAGATG
novel_mir31	CAAAACATATCCCAAATAAAATCCAAATCA	novel_mir107	AGCAGTTAAGTACAAGGAGTCGTCGGTGT
novel_mir32	AAAAATGAGTTGATTTGGATTTTATTTGGG	novel_mir108	CAAACGGATTCCAAATTGGCTCATTTTTTG
novel_mir33	TCTTAGGGGATAAATGTTTGTTTTATGATT	novel_mir109	AACTTGTCAAGCTAGTGTCAAACAACGACT
novel_mir34	ATCCTTAGGGCTGATGTCAGCA	novel_mir110	TTCCATTGTTAATTGTCAGGAAGAGTATCT
novel_mir35	GCCGGTCGAGCCGAATACTTGGGT	novel_mir111	TCCGAATTGTCTAGACCCTTCTGTCAAAAT
novel_mir36	CTGAGAGACGAACTCGTCAGGGACC	novel_mir112	ATTTTGACAGAAGGGTCTAGACAATTC
novel_mir37	ATTGCTTCTAGAGCGAACCTTTTT	novel_mir113	TTGCGAGAACCCGAATGTGTACTGTCAGC
novel_mir38	TCCTCGAAGCTTTGACCGTTCTTTGACAA	novel_mir114	ACATGTAAGGTTTGTCGTGGTC
novel_mir39	AACGGTCAAAGCTTCGAGGATTCAGTCA	novel_mir115	TTAGCCGAGTATGACTTGCCTATA
novel_mir40	TCTGTTTTTATGTTATTTCGTACTGTACA	novel_mir116	GCAGATCTTGGTGGTAGTAGCAAATATTCA
novel_mir41	TCGCCGCGCCTGCGGCGCGTCC	novel_mir117	ATTTGAGTTTGAAATCAACATGGTA

novel_mir42	GCGAACGTCGCCGCGCCTGCGG	novel_mir118	GTGCGGTCGCGCCGAAGGCGCGCG
novel_mir43	AACGGGTGCGAGCGCGCCTTC	novel_mir119	AAGAGCGATAGTACACGCCTAATTTAGGTA
novel_mir44	CATGGCACCTTTCGTTTCCAGCAATCCAAG	novel_mir120	AGACGAACAGAACATGATAGTTGTGTTGTG
novel_mir45	AGGTCTCTTTCTTTACTCT	novel_mir121	AACCGACCAAACCCAATAAC
novel_mir46	ACCGAATTCTGGACTAGATCTCCCCATTA	novel_mir122	TTGCCCCAGCCCGAAACCGACCGATA
novel_mir47	ACATTATCCGGCTTTACGTCATAGTGAATT	novel_mir123	CGTCTCGTCTGCACGGTGAAATATGCTATA
novel_mir48	TCCAGCTTGGATCGGAGAAAATCT	novel_mir124	TCGAAGCATGCAAAGTTCGCCGTTG
novel_mir49	GACCATTCACTATTTCTTGAAGCTCGATCT	novel_mir125	GTCGTGCTGAACTCGGATT
novel_mir50	TGGACCGAGGAGAAAGGGGGGCTC	novel_mir126	ACACACGGATTACCATGCGCTCACGATATT
novel_mir51	CCATTGGCACAACTTTGTTCGAAAATTCCA	novel_mir127	TGCCGTGTACTTTAGTTCTAGA
novel_mir52	CGGCGTGGCGAAAACAACGACGTGCGAGGT	novel_mir128	CTACGACGACGACACACCACGC
novel_mir53	CCTAACCCATCTGACTCTTCATACCCG	novel_mir129	ATTTGGACGTCTCAATACTTGA
novel_mir54	TTAGGGTTGATGACGTAAGAATT	novel_mir130	TCCAGGTTTGCTGTTTTGGGAAAATTGTT
novel_mir55	ATTACGGCTTCGGGTCAAA	novel_mir131	GGCACGCTGCAGGCTGCCAGGGCGCGTGC
novel_mir56	CACCCCACACATCGGGGTCGCGA	novel_mir132	TCAAGGATAAATGACGTAGGTT
novel_mir57	CCCCGAAGTATCGTTTGAGTCTCG	novel_mir133	CTTGTTGCTGACCAGCTCGAGA
novel_mir58	GCATGACCTGCCTGGTGGGTT	novel_mir134	ATCAGAGCAACTTGAAAATAAG
novel_mir59	GGAGTGATAGTAAGCGCCCTTGGCCGTT	novel_mir135	CGGTTATCAAAGTCGATGGTCAA
novel_mir60	TCCTAGGCTGTGGTGATAC	novel_mir136	CACAAAGGTCACTGAGAACCCCGTTGCTCC
novel_mir61	TTTGGATTTGGCTCATTTTTTGTA	novel_mir137	TCAGTGGGTCCCATTGTAGCGTG
novel_mir62	GCTAATTTTTGGTAGATGTGATCTATACTA	novel_mir138	GGCCGCAGAGGACTGTATCTGG
novel_mir63	TGAAGTGTTTGCCTTCTTGAC	novel_mir139	AAAGTCTGATGGCAAATATCAACCAAG
novel_mir64	AGGAGGGAGCTGATTTACTGATGTCGCAAC	novel_mir140	ACTAATGTGTTCTTAATTAAAAAT
novel_mir65	CAAAAATGAGACAATTTGAAGGCCT	novel_mir141	ATTTTGACTCTTTTCTCGCTCTCTA
novel_mir66	TAGAGGTTCAGATAATACAATTT	novel_mir142	AAATTTTGGTCTCTGTTCGAACATAGGG
novel_mir67	CGTCTGATTTGCCTGGCAGGGATCTCAACG	novel_mir143	GATGATATTCGAAGGAGCGTCTTCTCTGTT
novel_mir68	GTCTGATTTGCCTGGCAGGGATCTCAACG	novel_mir144	GAATGATGTGTGACCGCTATGACTCCCATT
novel_mir69	TTGGCTATCATTCAGTATTGTGCGTACA	novel_mir145	TCTATCATCCAAACAGTCGGTACGATT
novel_mir70	TAAGTTGGTAATAACACGTGGATCCCTTTA	novel_mir146	GTCCAACTCCTGGTCAATTTATATC

novel_mir71	CCTTTTGTATATTCCATCCTTT	novel_mir147	GTGTCTGCCAGCACGGTCCGTG
novel_mir72	TGCAGCCTACGTATACACAACAGGA	novel_mir148	GCAATAGGAGTCTTTTTGGACGGATTT
novel_mir73	GTTCGTCGTTGCATGCATGCAA	novel_mir149	CGTCGTAACTTGCTTGGTCATCACA
novel_mir74	AGAGTGATGAGTGAAATGCGCGAGG	novel_mir150	GTCATTACTTGGAAAAGCTCCG
novel_mir75	TGGTGTCAGTGGTAGCAAGG	novel_mir151	TCGTGGCCTCTTCTTTCTGGGTTTTCACAT
novel_mir76	TGAGCGCATGACAATCCGTGTGTCAAT		

Supplementary Table S7 Known miRNAs expression of samples in different periods.

miRNA Name	R1_1	R1_2	R1_3	R2_1	R2_2	R2_3	R3_1	R3_2	R3_3
miR3630-3p_1	0.12	0.08	0.3	0.14	0.04	0	0.24	0	1.18
miR164b	9.27	11.29	8.73	9.77	4.75	7.62	5.07	4.28	9.48
miR398a-3p_1	7.97	1.91	23.81	0.81	6.26	6.6	0.24	0.29	0.44
miR828a_1	0.06	0.08	0	0.14	0.04	0.28	0.59	0	0
miR172d_1	0	0.08	0	0.9	0.23	1.02	0.53	0.19	6.24
miR156a_2	2.07	4.04	6.55	2.58	3.56	10.35	6.78	3.61	4.61
miR169e_3	26.74	0.76	28.57	0.18	0.15	15.19	3.36	0.24	14.84
miR319_1	5.9	9.39	12.6	1.9	1.93	9.67	29.2	0.96	8.11
miR6300	166.31	207.66	567.78	90.06	9.47	27.42	11.62	23.62	43.77
miR168a-5p	838.5	2052.7	705.96	1760.45	1639.9	1529.7	2693.12	4506.7	1818.37
miR319a	5.67	15.42	11.51	16.1	5.53	5.29	8.44	4.23	5.99
miR390e	97.94	94.63	65.47	5.93	0.89	9.22	0.83	3.46	3.49
miR166d-5p_1	103.55	172.78	192.34	153.93	58.05	147.7	169.32	747.84	85.62
miR166h-3p	163.89	321.75	242.63	503.72	196.71	271.27	216.58	195.61	513.57
miR319b-5p_1	1.12	2.9	1.79	0.63	0.15	1.19	2.36	0.34	1.31
miR396a-5p	80.05	190.48	191.44	1.9	2.9	1.08	162.77	1.92	1023.39
miR390.1	0.06	0.23	0.2	0.05	0.08	0.06	0	0	0.25
miR159b-3p	6400.4	8857.9	11403.2	8734.5	2930.7	7722.35	17588.3	8294.6	22943.3
miR408-3p	2.54	4.12	4.07	0.41	0.08	0.4	1.83	0.96	1.31
miR319a_1	343.83	715.38	316.23	328.76	143.07	419.76	1420.13	102.61	504.4
miR171a-3p	151.43	552.3	489.32	752.06	311.1	792.53	292.45	237.8	870.62
miR156h	0.06	0	0.1	0	0.12	0	0	0	0.12
miR5368	0.12	0.23	0.6	0.14	0.04	0.51	0.12	0.38	0.25
miR168_1	0.59	0.61	0.99	1.9	0.85	2.56	3.66	0.58	2.18
miR4995	1.12	2.82	1.39	1.04	0.5	3.81	0.83	7.26	3.24
miR319a-5p_4	0.59	0.46	0.6	0.14	0.23	0.11	0.59	0.05	0.19
miR169b-5p	2.89	37.78	19.84	1.13	0.35	1.02	4.19	2.31	1.56
miR164a_3	168.37	234.29	118.63	236.21	108.59	198.27	142.83	160.63	263.27
miR394a	10.51	9.92	24.4	20.4	8.62	18.04	21.42	5.24	28.43
miR390a-5p	310.71	669.67	300.36	261.22	252.94	402.07	39	34.59	185.57
miR166	4.6	11.45	15.08	30.94	17.58	13.09	22.77	23.86	23.38
miR319c_3	2.07	25.79	107.72	29.72	1.97	112.65	45.37	0.82	10.91
miR160b_1	200.31	346.32	195.51	494.81	549.97	377.38	160.18	142.69	1000.69
miR168a-3p	911.76	1395.5	826.38	746.81	600.63	1111.59	1042.96	548.14	756.75
miR166m_2	29.05	84.56	4.76	20.4	5.8	9.39	31.03	23.81	43.09
miR5523	0.12	0	0.3	0.05	0	0.17	0	0	0.06
miR396h	1.65	2.59	2.88	3.8	1.7	3.36	1.59	0.63	7.61
miR172c-3p	0	0	0.1	0.18	0.08	0.06	0.06	0	0.69
miR530_3	0.12	0.31	2.98	0	0.04	0.57	13.51	0.34	0.44
miR168	206.92	347.69	238.86	122	72.46	90.63	123.01	280.04	140.49
miR390d-3p	299.08	664.63	172	186.77	133.56	459.02	40.35	43.78	137.25
miR408b_1	4.43	3.59	4.56	0.32	0.08	0.23	1.18	0.72	1.31

miR5179	0	0.08	0	0.09	0	0	0.71	0	0
miR166a-3p	3392.0	8233.8	3316.22	18944.2	7625.2	12273.8	13732.5	9703.5	16539.6
miR166a	591.13	1170.8	1108.09	1580.96	882.05	1663	2125.39	1127.6	3906.68
miR319a-3p	97.53	198.8	12.5	93.41	40.54	13.83	334.46	36.42	146.04
miR166c	0	0.23	0	0.05	0.04	0	0	0.14	0.06
miR396b	31.58	58.15	4.56	381.14	173.25	337.89	5.96	131.09	136.62
miR477e	2.48	4.73	13.29	0.63	1.08	1.54	11.39	1.88	3.3
miR162-3p	42.33	72.27	45.03	49.26	23.5	99.34	85.78	16.21	75.33
miR169v_1	3.6	6.33	8.03	2.85	2.24	0.28	47.61	1.59	0.06
miR156k	0.18	0.38	0.2	0.05	0.12	0.17	0.06	0.05	0.06
miR166i_1	1.71	5.72	2.88	11.4	5.37	8.02	8.44	5.58	14.34
miR168b_1	8.91	19.99	16.96	15.33	14.53	10.53	13.63	116.18	23.76
miR166e	15.76	3.51	44.14	48.49	17.78	32.66	12.57	2.02	7.42
miR5021	0	0	0	0	0	0.11	0	0	0
miR396g	0	0	0	0	0	0	0	0	0.06
miR160b_1	0	0	0	0	0	0	0	0	0.06

Supplementary Table S8 Novel miRNAs expression of samples in different periods.

novel miRNA	R1-1	R1-2	R1-3	R2-1	R2-2	R2-3	R3-1	R3-2	R3-3
novel_mir1	34.36	59.68	36.21	1.58	0	0	0	1.59	0
novel_mir2	1.24	2.14	0	0	0	0	0	0	0
novel_mir3	5.14	13.05	7.34	14.75	9.7	44.04	86.31	49.84	55.87
novel_mir4	181.77	203.99	242.82	562.98	24	327.31	608.27	309.86	199.85
novel_mir5	55.67	83.64	79.26	103.81	73.04	169.32	327.85	99.01	272.12
novel_mir6	365.2	512.38	625.51	286.19	25	639.71	291.45	872.53	59.99
novel_mir7	4.37	8.24	9.52	9.32	5.8	17.07	33.86	85.01	13.34
novel_mir8	735.6	1006.15	944.32	867.36	532.27	1198.98	1823.73	1209.29	1478.28
novel_mir9	11.81	15.19	20.63	6.6	2.13	2.96	9.2	9.67	23.13
novel_mir10	25.21	14.12	26.98	12.03	0	7.68	3.24	19.39	0.81
novel_mir11	34.36	69.37	52.47	60.66	31.15	100.36	139.41	84.96	109.06
novel_mir12	35.72	79.14	55.94	62.33	38.45	83.06	130.8	68.99	79.13
novel_mir13	7.67	15.8	9.13	14.07	7.3	21.51	35.16	47.63	28.68
novel_mir14	146.71	219.33	178.15	443.56	277.4	479.61	348.68	258.48	687.91
novel_mir15	31.17	48.84	43.64	35.87	16.77	70.26	110.74	71.15	86.11
novel_mir16	3.01	2.75	0	1.67	1.85	9.79	5.96	1.01	0.75
novel_mir17	60.81	76.39	90.86	1.27	0	0	0	4.96	0.69
novel_mir18	40.03	54.72	50.39	56.18	19.4	46.37	59.88	47.05	42.53
novel_mir19	68.01	0.84	0	1.18	0	2.16	14.22	10.15	2.12
novel_mir20	17.18	102.87	62	46.68	30.57	54.67	188.14	49.6	14.22
novel_mir21	195.77		8.73	12.44	13.8	1.37		9.96	47.7
novel_mir22	326.35	195.9	209.99	56.45	33.43	75.38	120.41	39.21	64.98
novel_mir23	0	0	0	0.5	0	0	0	0	0.94
novel_mir24	2410.41	763.84	718.85	733.56	400.29	906.37	1343.02	976.25	896.93
novel_mir25	462.02	3374.22	3965.04	4275.99	2220.12	4099.7	5845.01	6082.82	10103.94
novel_mir26	25.09	499.64	586.93	708.95	426.8	608.99	398.12	886.87	551.85
novel_mir27	4.25	16.64	30.85	13.57	8	8.7	5.96	9.33	0.81
novel_mir28	779.76	7.71	6.25	7.83	2.98	13.65	34.75	19.92	23.45
novel_mir29	4.01	600.6	437.14	8.37	1.24	0.8	9.14	29.68	16.21
novel_mir30	8.74	6.87	5.06	10.54	6.03	12.52	31.5	15.64	
novel_mir31	65.53	21.67	18.65	80.47	56.62	80.33	29.97	21.46	102.26
novel_mir32	0	0	0	0	0	0	1	0	0
novel_mir33	90.86	25.03	6.25	0	0	0	0	0	0
novel_mir34	1.77	110.05	217.63	29.54	16.54	45.4	60.41	59.85	71.9
novel_mir35	6.61	9.46	3.47	14.29	7.07	24.58	31.8	12.6	25.69
novel_mir36	4.96	16.41	6.84	19.68	12.33	36.07	62.01	18.09	28.56
novel_mir37	14.46	17.55	4.27	16.28	4.56	28.79	43.19	22.61	36.6
novel_mir38	112.17	33.88	12.6	39.44	20.87	74.47	119.06	58.4	77.7
novel_mir39	0	0	0	3.75	0	4.49	3.95	1.01	0.75

novel_mir40	41.21	149.96	164.16	263.58	37.72	325.77	1322.02	284.56	122.03
novel_mir41	30.52	59.6	51.08	80.79	31.19	88.87	129.44	46.42	61.48
novel_mir42	171.56	41.9	27.58	52.34	24.42	66.57	121.36	73.32	51.69
novel_mir43	0	0	0	0.9	0	0.74	3.36	1.3	0
novel_mir44	0	0	0	1.4	0.77	2.45	7.91	1.2	14.47
novel_mir45	36.48	266.8	171.01	13.07	1.85	7.74	13.16	14.67	33.05
novel_mir46	4353.66	53.96	50.39	47.09	57.93	53.82	92.57	21.74	1.25
novel_mir47	23.08	6851.24	6686.9	6885.3	6244.54	6004.96	5639.17	8855.86	11881.53
novel_mir48	3.78	65.71	59.32	59.66	70.8	83.29	168.2	38.39	67.22
novel_mir49	1.71	16.1	0	1.49	0.39	51.66	1.47	23.14	5.49
novel_mir50	11.45	9.46	3.07	0.5	0	16.16	0	0.53	2.31
novel_mir51	366.15	28.08	20.93	24.92	10.86	59.85	62.18	36.85	48.64
novel_mir52	23.97	897.01	907.72	705.19	342.6	902.9	987.8	710.84	779.7
novel_mir53	2.07	40.52	37.2	38.22	25.47	78.11	74.81	26.84	158.57
novel_mir54	88.79	3.21	3.77	13.3	3.83	7.51	18.88	9.53	6.67
novel_mir55	5.73	140.88	130.44	157.59	92.52	248.8	359.94	179.2	273.43
novel_mir56	423.82	20.61	9.03	59.3	39.77	44.15	21.24	57.87	48.76
novel_mir57	37.49	743.85	1016.83	455.41	225.11	818.53	1014.35	2163.37	373.83
novel_mir58	57.56	62.5	63.48	68.57	54.8	60.14	116.64	34.59	104.88
novel_mir59	0	0	0	0	0.39	0.57	1.89	1.97	1.81
novel_mir60	29.11	80.21	1641.05	896.49	70.8	1656.4	1048.45	2944.59	0
novel_mir61	12.04	46.86	40.27	44.46	23.73	72.2	137.82	70.33	101.02
novel_mir62	554.65	17.55	15.18	34.15	16.31	48.47	81.95	24.82	65.35
novel_mir63	570.35	1035.07	785.31	605.54	460.66	716.06	1115.47	580.23	524.54
novel_mir64	26.27	896.71	1023.77	396.29	317.59	493.44	802.19	470.88	385.3
novel_mir65	58.39	26.25	35.91	36.59	18.86	57.75	115.16	45.94	93.04
novel_mir66	5453.22	186.44	174.78	247.97	96.11	299.49	617.71	87.32	121.47
novel_mir67	27.33	9270.83	6376.72	6499.09	4122.97	13669.78	31853.34	5941.96	6947.97
novel_mir68	429.37	26.48	54.85	52.7	45.1	71.06	188.97	63.5	0
novel_mir69	75.63	613.58	663.8	829.59	513.18	799.64	1052.28	1171.77	1300.31
novel_mir70	152.26	140.12	174.28	129.55	57.85	143.54	299.71	180.12	285.28
novel_mir71	1501.54	234.52	319.1	241.05	124.28	273.2	537.82	171.41	569.19
novel_mir72	11.22	2604.88	3344.19	3733.87	1860.72	3465.28	7969.22	1416.97	4517.27
novel_mir73	65	14.65	15.87	20.49	38.65	28.96	38.7	25.55	30.12
novel_mir74	0.83	105.54	119.13	106.21	76.6	187.52	220.71	146.97	157.08
novel_mir75	1371.25	1.6	2.98	4.3	4.21	10.98	24.48	26.27	51.13
novel_mir76	183.66	2531.39	2612.45	1654.24	1037.14	2923.2	3755.49	768.67	1618.7
novel_mir77	18.36	516.43	233.2	884.46	3024.96	465.62	457.59	428.88	733
novel_mir78	3.78	31.59	34.82	29.9	10.59	45.17	65.96	36.75	28.31
novel_mir79	40.79	3.89	1.19	2.22	0.43	2.33	9.38	20.83	2.24
novel_mir80	17.53	77.54	66.46	83.37	35.4	142.58	174.28	114.11	104.07
novel_mir81	12.69	42.2	24.2	25.92	18.36	34.25	52.21	17.61	53.19
novel_mir82	0.89	26.86	14.48	45.05	20.06	87.5	101.3	54.22	41.84
novel_mir83	8.15	2.59	0.99	2.04	3.63	3.13	3.36	4.67	6.8
novel_mir84	985.38	39.61	39.78	36.91	17.04	21.51	72.15	6.49	53.38
novel_mir85	64.35	615.18	634.74	46.05	22.88	117.49	586.38	25.79	40.53
novel_mir86	2.18	118.9	144.43	94.22	77.56	99.51	154.4	120.22	201.41
novel_mir87	2.36	2.06	2.88	2.04	1.28	6.26	6.73	0	0
novel_mir88	21.37	3.89	2.28	2.22	0.93	5.69	25.07	5.63	10.6
novel_mir89	0.65	65.86	55.94	81.01	60.36	90.8	157.58	94.39	193.8
novel_mir90	2.07	0.92	3.07	3.48	0.7	3.07	7.96	3.56	5.92
novel_mir91	142.75	2.14	0.99	4.61	0	7.05	17.35	22.03	5.18
novel_mir92	0	133.09	243.62	252.04	249.03	367.02	389.74	182.33	534.14
novel_mir93	12.63	2.82	0	4.43	5.26	7.57	6.49	4.09	3.24
novel_mir94	179.94	9.84	10.71	8.37	2.78	43.41	45.55	26.75	44.02
novel_mir95	0.83	95.09	26.58	44.87	29.18	255	15.46	90.2	175.35
novel_mir96	23.91	2.44	4.66	5.25	0.5	7.28	14.57	6.11	5.99
novel_mir97	9.62	46.17	76.18	24.7	6.42	17.69	41.06	10.25	51.32
novel_mir98	6.67	49.53	14.09	23.75	19.71	49.84	13.45	21.46	114.55
novel_mir99	29.05	10	6.84	16.47	4.48	19.8	41.24	18.76	30.24
novel_mir100	4.96	14.04	20.83	0.45	0	0	2.12	0	2

novel_mir101	4.84	19.31	33.53	24.79	2.32	64.63	3.01	14.87	0
novel_mir102	125.63	15.03	7.34	12.98	4.41	26.34	36.81	31.22	26
novel_mir103	61.22	204.53	280.52	231.23	136.27	215.29	568.03	210.47	556.28
novel_mir104	29.22	90.59	84.51	123.8	60.52	162.83	240.36	132.3	222.49
novel_mir105	17.77	35.03	25.69	33.25	40.96	44.43	826.2	48.16	383.3
novel_mir106	73.91	17.55	17.76		0.39	4.32	4.31	0.53	6.3
novel_mir107	4.96	156.14	116.25	132.26	65.39	250.56	311.57	255.74	229.72
novel_mir108	0	0	0	0	0	0	0	0	2.81
novel_mir109	0	7.56	4.07	10.81	5.41	26.23	39.53	18.18	21.08
novel_mir110	2.42	0.76	0	3.53	0	0	0	0	18.89
novel_mir111	26.74	2.21	3.27	2.4	1.24	5.06	6.84	1.78	5.99
novel_mir112	41.74	71.28	53.17	117.56	107.63	155.72	63.89	90.59	178.46
novel_mir113	32.59	102.03	113.77	97.52	49.23	191.39	284.13	157.46	218.37
novel_mir114	46.82	46.55	72.31	35.24	23.96	66.79	93.63	87.46	70.59
novel_mir115	99.36	81.12	292.42	12.48	13.06	111.06	234.99	132.25	352.56
novel_mir116	11.57	55.63	114.47	102.73	74.93	128.07	107.85	170.25	375.63
novel_mir117	15.11	13.51	9.03	23.52	9.74	43.47	65.07	27.81	40.16
novel_mir118	79.94	32.28	20.04	35.83	21.18	47.11	56.05	26.22	44.09
novel_mir119	17.36	143.32	103.56	171.57	51.79	247.72	415.23	164.38	253.35
novel_mir120	2.42	90.89	474.54	262.04	769.21	210.68	46.43	142.69	443.91
novel_mir121	37.96	7.78	2.58	10.31	3.4	21.68	42.77	17.56	24.51
novel_mir122	0	0	0	0.59	0	0.91	2.71	2.5	1.37
novel_mir123	0.71	80.51	62	54.82	51.01	74.08	115.99	72.5	48.82
novel_mir124	0	0	0	0.68	0.39	0.8	5.43	0.48	1.31
novel_mir125	264.9	1.68	0	1.63	1.7	4.72	8.5	15.83	4.36
novel_mir126	157.16	402.79	540.21	142.21	48	398.82	734.82	204.84	166.55
novel_mir127	0	322.43	457.97	608.03	357.59	445.7	131.68	201.96	513.32
novel_mir128	27.57	0.84	0	0	0	1.48	3.3	0.48	1.37
novel_mir129	212.36	45.56	46.92	41.03	28.02	90.92	116.52	57.3	90.79
novel_mir130	52.78	408.21	309.28	353.59	113.85	433.7	279.88	119.07	154.02
novel_mir131	0	90.51	69.44	81.56	36.67	111.74	115.87	57.15	91.98
novel_mir132	1.59	0	2.28	0	0	0	29.85	0	0
novel_mir133	2.6	6.79	3.87	7.06	2.86	13.26	22.48	18.67	15.09
novel_mir134	1.48	5.49	5.16	4.61	1.89	7.85	18.47	1.35	9.1
novel_mir135	0	0	0	8.28	64.54	0	0	0.48	0
novel_mir136	2.77	4.96	3.87	2.8	1.78	7.74	9.03	11.5	3.8
novel_mir137		4.27	1.09	2.71	1.47	8.19	8.91	13.42	4.61
novel_mir138	1.36	1.6		2.76	0.46	8.7	10.68	5.2	8.85
novel_mir139		1.76	0.99	4.39	0.5	7.4	14.93	8.8	7.42
novel_mir140	37.96	0	0	2.94	2.86	5.29	15.04	1.92	4.43
novel_mir141	0.89	43.12	23.71	28.45	10.63	52.34	55.87	19.29	23.07
novel_mir142	35.42	2.06	0.99	1.85	0	4.27	15.58	7.84	10.29
novel_mir143	2.3	44.72	42.65	46.86	10.51	37.32	43.95	35.94	40.47
novel_mir144	9.5	3.97	0.99	5.38	1.28	11.26	24.31	19	10.04
novel_mir145	8.09	17.71	18.85	27.55	16.19	38	73.69	115.75	143.23
novel_mir146	4.49	33.5	37.69	73.28	56.81	58.71	8.67	16.65	58.24
novel_mir147	1.42	13.89	4.66	17.28	5.37	29.3	44.84	19.58	27
novel_mir148	23.67	1.14	3.37	2.04	1.28	4.89	5.37	2.79	3.8
novel_mir149	0.77	40.98	35.21	54.33	14.76	115.32	78.05	135.86	90.04
novel_mir150	112.46	0.76	0	0.95	0	2.79	0	0.48	7.17
novel_mir151	0	224.37	202.35	131.18	66.47	181.09	446.02	844.39	0

Supplementary Table S9 Target genes of 7 novel miRNAs.

miRNA id	Target id	Pathway
novel_mir1	CL10132.Contig6_All	-
novel_mir1	CL8972.Contig1_All	-
novel_mir1	CL7498.Contig2_All	K00020|1|3.0e-12|75.1|pop:POPTR_0015s04730g|3-hydroxyisobutyrate dehydrogenase [EC:1.1.1.31]
novel_mir2	CL93.Contig2_All	K13457|1|5.2e-10|68.6|mdm:103433497|disease resistance protein RPM1
novel_mir2	CL224.Contig2_All	K16190|1|1.4e-21|106.7|bdi:100843665|glucuronokinase [EC:2.7.1.43]!K13422|2|7.8e-20|100.9|jcu:105637396|transcription factor MYC2
novel_mir2	CL93.Contig3_All	K13457|1|3.4e-24|115.9|mdm:103433497|disease resistance protein RPM1
novel_mir2	Unigene17168_All	K02882|1|1.8e-71|272.3|adu:107496420|large subunit ribosomal protein L18Ae!K01381|3|4.6e-43|177.9|smo:SELMODRAFT_13348|saccharopepsin [EC:3.4.23.25]
novel_mir2	CL93.Contig5_All	K13457|1|2.1e-24|116.3|mdm:103433497|disease resistance protein RPM1
novel_mir16	CL351.Contig4_All	K13428|1|1.2e-192|675.6|brp:103856446|LRR receptor-like serine/threonine-protein kinase EFR [EC:2.7.11.1]
novel_mir16	Unigene34399_All	K13412|1|2.0e-24|115.9|pmum:103340207|calcium-dependent protein kinase [EC:2.7.11.1]
novel_mir16	Unigene19617_All	K14491|1|3.5e-230|800.0|nnu:104610687|two-component response regulator ARR-B family
novel_mir16	Unigene71964_All	-
novel_mir16	Unigene23183_All	K04733|1|4.3e-80|300.8|cit:102607371|interleukin-1 receptor-associated kinase 4 [EC:2.7.11.1]
novel_mir16	CL351.Contig7_All	K13428|1|1.3e-228|795.0|bna:106407525|LRR receptor-like serine/threonine-protein kinase EFR [EC:2.7.11.1]
novel_mir16	Unigene13443_All	K04730|1|3.0e-108|394.0|rcu:8262706|interleukin-1 receptor-associated kinase 1 [EC:2.7.11.1]
novel_mir16	Unigene13451_All	K04730|1|5.2e-42|172.9|cic:CICLE_v10011359mg|interleukin-1 receptor-associated kinase 1 [EC:2.7.11.1]
novel_mir16	Unigene34397_All	K13412|1|2.4e-24|115.9|pper:PRUPE_ppa003676mg|calcium-dependent protein kinase [EC:2.7.11.1]
novel_mir16	Unigene13446_All	K04730|1|7.6e-109|396.0|rcu:8262706|interleukin-1 receptor-associated kinase 1 [EC:2.7.11.1]
novel_mir16	CL4656.Contig3_All	K03086|1|4.7e-197|690.6|pmum:103339111|RNA polymerase primary sigma factor

novel_mir16	CL12635.Contig9_All	K10268|1|3.1e-23|111.3|ats:F775_00106|F-box and leucine-rich repeat protein 2/20!K11446|2|1.7e-21|105.5|mdm:103446667|histone demethylase JARID1 [EC:1.14.11.-]!K13336|3|8.4e-21|103.2|gra:105801020|peroxin-3!K15923|5|1.1e-17|92.8|ats:F775_11819|alpha-L-fucosidase 2 [EC:3.2.1.51]
novel_mir16	Unigene4656_All	K00011|1|8.2e-87|323.2|egr:104426746|aldehyde reductase [EC:1.1.1.21]!K01535|2|1.3e-76|289.3|obr:102712647|H+-transporting ATPase [EC:3.6.3.6]
novel_mir16	CL2081.Contig3_All	K10436|1|8.0e-115|415.6|tcc:TCM_013936|microtubule-associated protein, RP/EB family
novel_mir16	Unigene34895_All	K10581|1|0.0e+00|1402.5|nnu:104604279|ubiquitin-conjugating enzyme E2 O [EC:2.3.2.24]
novel_mir16	Unigene28796_All	K01889|1|1.8e-06|56.6|tcc:TCM_020258|phenylalanyl-tRNA synthetase alpha chain [EC:6.1.1.20]!K08955|2|3.1e-06|55.8|mtr:MTR_5g074850|ATP-dependent metalloprotease [EC:3.4.24.-]
novel_mir16	Unigene34408_All	K13412|1|8.3e-130|465.7|aip:107623623|calcium-dependent protein kinase [EC:2.7.11.1]
novel_mir16	CL351.Contig5_All	K13428|1|1.9e-198|694.9|brp:103856446|LRR receptor-like serine/threonine-protein kinase EFR [EC:2.7.11.1]
novel_mir16	CL4935.Contig1_All	K11262|1|0.0e+00|1884.0|nnu:104605928|acetyl-CoA carboxylase / biotin carboxylase 1 [EC:6.4.1.2 6.3.4.14]
novel_mir16	Unigene13450_All	K04730|1|1.4e-47|192.2|nnu:104606348|interleukin-1 receptor-associated kinase 1 [EC:2.7.11.1]
novel_mir16	CL237.Contig2_All	K13459|1|4.2e-100|368.2|nnu:104592365|disease resistance protein RPS2
novel_mir16	CL351.Contig9_All	K13428|1|2.0e-203|711.4|brp:103856446|LRR receptor-like serine/threonine-protein kinase EFR [EC:2.7.11.1]
novel_mir16	CL5184.Contig2_All	K13448|1|6.2e-56|219.9|vvi:100245811|calcium-binding protein CML
novel_mir16	CL12114.Contig3_All	K10706|1|1.3e-62|243.8|cit:102613717|senataxin [EC:3.6.4.-]
novel_mir16	Unigene5597_All	K03283|1|0.0e+00|1225.3|jcu:105636278|heat shock 70kDa protein 1/8
novel_mir16	Unigene3402_All	K15923|1|2.7e-23|112.1|ats:F775_11819|alpha-L-fucosidase 2 [EC:3.2.1.51]!K10268|2|4.6e-23|111.3|ats:F775_00106|F-box and leucine-rich repeat protein 2/20!K11446|3|2.5e-21|105.5|mdm:103446667|histone

		demethylase JARID1 [EC:1.14.11.-]!K13336|4|1.3e-20|103.2|gra:105801020|peroxin-3
novel_mir16	CL12559.Contig5_All	K16196|1|8.9e-35|149.8|pda:103708605|eukaryotic translation initiation factor 2-alpha kinase 4 [EC:2.7.11.1]!K15923|2|1.7e-30|135.6|ats:F775_11819|alpha-L-fucosidase 2 [EC:3.2.1.51]!K00963|4|7.3e-13|77.0|gra:105775510|UTP--glucose-1-phosphate uridylyltransferase [EC:2.7.7.9]!K10268|5|9.5e-13|76.6|ats:F775_00106|F-box and leucine-rich repeat protein 2/20
novel_mir23	Unigene22505_All	K14509|1|8.9e-288|991.1|vvi:100241730|ethylene receptor [EC:2.7.13.-]
novel_mir101	Unigene22949_All	K11808|1|9.8e-280|964.5|nnu:104589619|phosphoribosylaminoimidazole carboxylase [EC:4.1.1.21]
novel_mir101	CL2086.Contig3_All	K12176|1|3.0e-242|839.3|nnu:104593548|COP9 signalosome complex subunit 2
novel_mir101	CL1164.Contig1_All	K00327|1|5.0e-159|562.4|vvi:100240979|NADPH-ferrihemoprotein reductase [EC:1.6.2.4]
novel_mir102	Unigene8546_All	-
novel_mir102	CL12209.Contig1_All	K08247|1|3.0e-80|302.8|thj:104800334|methionine S-methyltransferase [EC:2.1.1.12]!K01191|2|6.5e-67|258.5|pda:103707976|alpha-mannosidase [EC:3.2.1.24]!K15255|3|1.5e-39|167.5|mtr:MTR_0003s0410|ATP-dependent DNA helicase PIF1 [EC:3.6.4.12]!K05666|4|7.7e-36|155.2|cam:101504903|ATP-binding cassette, subfamily C (CFTR/MRP), member 2
novel_mir102	CL10854.Contig10_All	K01303|1|3.8e-111|407.1|fve:101311565|acylaminoacyl-peptidase [EC:3.4.19.1]!K14638|4|1.0e-31|143.3|bna:106412703|solute carrier family 15 (peptide/histidine transporter), member 3/4!K07478|5|3.0e-31|141.7|egr:108958286|putative ATPase
novel_mir102	Unigene9769_All	K17265|1|2.4e-60|236.5|nnu:104609434|Ras GTPase-activating protein-binding protein 1 [EC:3.6.4.12 3.6.4.13]
novel_mir121	CL10252.Contig3_All	-
novel_mir121	Unigene13827_All	K04730|1|2.9e-06|55.1|bvg:104902207|interleukin-1 receptor-associated kinase 1 [EC:2.7.11.1]
novel_mir121	Unigene12383_All	K18757|1|1.1e-52|210.7|gmx:100780505|la-related protein 1
novel_mir121	CL337.Contig5_All	K18080|1|8.9e-151|536.6|vvi:109124306|tensin!K02184

novel_mir121	Unigene40939_All	|4|2.1e-147|525.4|cam:101505448|formin 2 K03327|1|1.0e-42|174.5|vvi:100248568|multidrug resistance protein, MATE family

Supplementary Table S10 GO functional classification of target genes regulated by miRNAs.

GO Classification	GO Group	Gene Count
biological_process	biological regulation	26
	cellular component organization or biogenesis	11
	cellular process	82
	developmental process	7
	localization	15
	metabolic process	74
	multi-organism process	1
	multicellular organismal process	7
	negative regulation of biological process	5
	positive regulation of biological process	2
	regulation of biological process	24
	reproductive process	1
	response to stimulus	13
	signaling	8
	single-organism process	55
cellular_component	cell	85
	cell part	85
	extracellular region	2
	macromolecular complex	16
	membrane	65
	membrane part	59
	membrane-enclosed lumen	2
	organelle	56
	organelle part	21
molecular_function	binding	108
	catalytic activity	92
	electron carrier activity	2
	enzyme regulator activity	1
	guanyl-nucleotide exchange factor activity	1
	molecular transducer activity	5
	nucleic acid binding transcription factor activity	14
	protein binding transcription factor activity	1
	receptor activity	4
	structural molecule activity	4
	transporter activity	11

Supplementary Table S11 KEGG functional classification of target genes regulated by miRNAs.

Pathway level1	Pathway level2	Number of Genes
Cellular Processes	Transport and catabolism	10
Environmental Information Processing	Membrane transport	8
	Signal transduction	11
Genetic Information Processing	Folding, sorting and	22
	Replication and repair	5
	Transcription	10
	Translation	11
Metabolism	Amino acid metabolism	11
	Biosynthesis of other secondary	8
	Carbohydrate metabolism	20
	Energy metabolism	6
	Global and overview maps	48
	Glycan biosynthesis and	6
	Lipid metabolism	7
	Metabolism of cofactors and	5
	Metabolism of other amino	9
	Metabolism of terpenoids and	5
	Nucleotide metabolism	5
Organismal Systems	Environmental adaptation	18
